# Light does not phase shift the circadian clock of subcutaneous adipose tissue in vitro

**DOI:** 10.1038/s44323-025-00048-y

**Published:** 2025-09-04

**Authors:** Anna Edmondson, Charley Beresford, Jan-Frieder Harmsen

**Affiliations:** 1 MRC Laboratory of Molecular Biology Cambridge, Cambridge, UK; 2 Healthy Living Spaces Lab, Institute for Occupational, Social and Environmental Medicine, Medical Faculty, RWTH Aachen University, Aachen, Germany; 3 Chair of Healthy Living Spaces, Faculty of Architecture, RWTH Aachen University, Aachen, Germany; 4 Department of Nutrition and Movement Sciences, NUTRIM Institute of Nutrition and Translational Research in Metabolism, Maastricht University Medical Center, Maastricht, The Netherlands

**Keywords:** Circadian rhythms, Visual system

## Abstract

The retinal photopigment melanopsin is also expressed in subcutaneous white adipose tissue (scWAT). Through melanopsin, light can modulate scWAT metabolism, but its impact on circadian phase is unclear. In vitro exposure of murine scWAT to bright light at different times over 24 h did not elicit phase shifts, unlike the response to corticosterone. This finding suggests that the direct impact of bright light on scWAT metabolism occurs in a circadian-independent manner.

Besides enabling visual perception, ocular light exposure affects various aspects of human physiology and behavior, such as circadian rhythms, sleep, neuroendocrine activity, mood, and cognitive function^1–5^. Light aligns mammalian circadian rhythms with the external environment by adjusting signals predominantly from intrinsically photosensitive retinal ganglion cells (ipRGCs) that reach the central circadian pacemaker, the suprachiasmatic nucleus (SCN) in the hypothalamus^6,7^. These ipRGCs contain a light-sensing photopigment called melanopsin, which is maximally sensitive to the blue color spectrum of light (e.g., ≈480 nm)^8,9^. Circadian photoentrainment is thought to occur in a hierarchical manner, with photic input to the retina exclusively entraining the SCN, which subsequently synchronizes peripheral tissue clocks via endocrine mechanisms. Intriguingly, even blind humans can show circadian photoentrainment of the SCN^10^ as long as ipRGCs are intact, since extraocular phototransduction was not found to phase shift enucleated mammals, as shown in hamsters^11^. Nonetheless, extraocular photoentrainment of the SCN has been proposed to occur in humans^12^ but could not be replicated since^13–15^.

However, the gene *Opn4* encoding melanopsin is also expressed in other mammalian tissues, where it may not necessarily serve circadian entrainment functions. For example, blue light has been found to induce subcutaneous vasorelaxation through activation of melanopsin located in blood vessels^16^. Interestingly, *Opn4* is also highly expressed in human subcutaneous white adipose tissue (scWAT), but not visceral WAT^17^. Using mouse retina as a positive control, Ondrusova et al. observed the presence of *Opn4* mRNA in human scWAT and murine differentiated adipocytes, identifying a light-sensitive signaling pathway mediated via a melanopsin/TRPC channel axis^17^. Testing the physiological relevance of this pathway, they showed that exposing differentiated adipocytes to blue light for 4-h daily over a period of 13 days led to a reduction in lipid droplet size, an increase in basal lipolysis, and changes in adiponectin and leptin secretion^17^. Ondrusova et al. further speculated that proper sunlight exposure to scWAT may function as a peripheral circadian sensor, supporting overall metabolic health. While more studies have identified other roles of opsins (e.g., opsin3) in adipocyte metabolism^18,19^, the possibility that blue light may exert metabolic effects in scWATs by inducing circadian phase shifts of the adipocyte molecular clock has, to the best of our knowledge, not yet been thoroughly investigated.

Therefore, in the present study, we investigated whether bright light exposure can reset the circadian phase of murine scWAT in vitro. Initially, we monitored the circadian phase of both subcutaneous fat explants and fibroblasts from PER2::LUC mice by measuring PER2::LUCIFERASE expression via bioluminescence. Fibroblasts were used as a negative control, as it has been demonstrated previously that fibroblasts do not respond to light^20^. Fat explants and fibroblasts were both treated with the natural rodent glucocorticoid (e.g., corticosterone; CORT), as a positive control, as CORT is known to potently reset the phase of cells^21–23^, or treated with a 4-hour continuous bright light stimulus. The light source emitted a broad wavelength spectrum (Fig. 1A), with a peak emission at 450 nm, an intensity of 1800 photopic lux, and a correlated color temperature (CCT) of 5000 K (relative spectral power distribution in Fig. 1B). Concurrently, a vehicle (DMSO) as well as an untreated condition served as negative controls. The untreated, DMSO and CORT-treated tissues/fibroblasts were not exposed to bright light after the onset of bioluminescence acquisition, and treatments were conducted under minimal light.

**Fig. 1:**
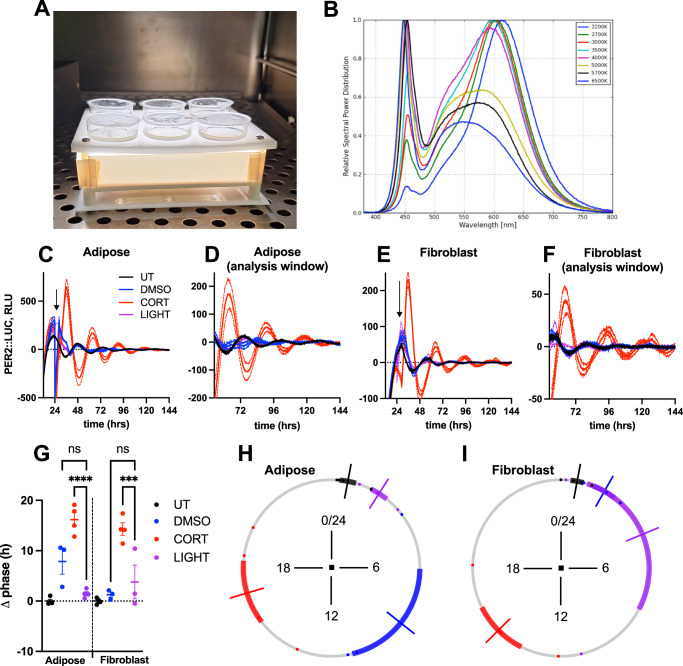
Comparative phase-shift responses of adipose tissue and fibroblasts to bright light exposure and corticosterone. **A** Incubation set-up of the cell cultures on top of the white light box. **B** The relative spectral power distribution of the white light box was constantly set to a correlated color temperature of 5000 K. **C** Detrended bioluminescence trace of adipose tissue explant from PER2::LUC mice treated with either vehicle (DMSO), 100 nM corticosterone (CORT) or 4–h incubation on a white light box (LIGHT), measured in relative luminescence units (RLU), *n* ≥ 4 biological replicates (dishes of tissue from 5 mice), mean ± SEM. UT= untreated. **D** Data from **A**, used during quantitative phase analysis in (**G**). Detrended bioluminescence trace of PER2::LUC mouse fibroblasts treated with either DMSO, CORT, or LIGHT measured in RLU, *n* = 4 biological replicates (dishes of fibroblasts), mean ± SEM. **F** Data from **C**, used during quantitative phase analysis in (**E**). **G** Quantification of treatment-induced change (Δ) in phase (h) from (**B**) and (**D**), *n* = 4, mean ± SEM, one-way ANOVA (OWA), Holm–Sidak’s MCT, ****= *p* < 0.0001, *** = *p* = 0.001, ns = *p* > 0.05. **H**, **I** Circular plots depicting quantification of treatment-induced change in phase (h) from (**D**) and (**F**), respectively. black = untreated, blue = DMSO, red = CORT, purple = LIGHT. Hours are plotted around the circle axis, the mean of each condition is depicted by colored lines, and SEM is the length of the colored bar around the circle.

The detrended bioluminescence traces of adipose tissue explant and fibroblasts from PER2::LUC mice following treatment, as well as the resulting quantification of phase shifts, are shown in Fig. 1. As expected, CORT consistently reset the circadian phase of both scWAT (16.2 ± 2.8 h vs. 7.9 ± 4.4 h, *p* = 0.0109, Fig. 1G, H) and fibroblasts (mean ± SEM; 14.3 ± 2.5 h vs. 1.3 ± 0.9, *p* < 0.0001, Fig. 1G, I) compared to the vehicle (DMSO) condition. However, white light exposure did not induce any significant phase shifts, for both scWAT (1.5 ± 0.8 h, Fig. 1G, H) and fibroblasts (3.8 ± 5.8 h, Fig. 1G, I) compared to the vehicle (DMSO) condition. Following CORT, the circadian phase of both scWAT (*p* < 0.0001, Fig. 1G, H) and fibroblasts (*p* = 0.001, Fig. 1G, I) significantly differed from the respective white light treatment condition.

However, the most accurate way to quantify the phase dependence of light-induced phase shifts is through the construction of a phase response curve (PRC), which systematically evaluates the response to a defined light stimulus across the full circadian cycle^5^. The precise shape and timing of the resulting PRC yield detailed insights into the relationship between the magnitude of the phase shift and the circadian phase at which the stimulus occurs. As the 4-h bright light stimulus of the first experiments was applied only at a single time point (e.g., 31 h after the last media change), we next performed a more comprehensive set of experiments around the clock. Accordingly, the 4-h bright light stimulus was applied at 25, 31, 37, and 43 h after the last media change. An additional control condition of incubation in darkness was also conducted. In these PRC-yielding experiments, we also added 1 µM all-trans-retinal to each scWAT explant culture, as supplementing retinaldehyde was found to be required to elicit a light response in adipocytes^17^, and its absence in the initial experiments could have also explained the absence of a light-induced phase shift. The detrended bioluminescence traces of adipose tissue explant from PER2::LUC mice following white light treatment at different phases of the circadian cycle as well as the resulting quantification of phase shifts are shown in Fig. 2. As expected, the 4-hour bright light stimulus did not induce a shift in the circadian phase at any time from 25 to 43 h after the last media change, similarly to 4-h darkness (one-way ANOVA treatment effect: *p* = 0.933, Fig. 2B). With no significant phase shifts, bright light hence yielded a flat PRC (Fig. 2C).

**Fig. 2:**
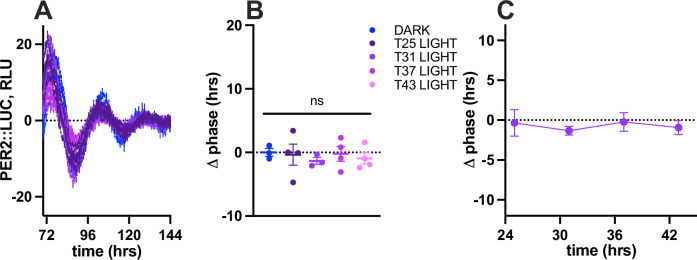
Phase-response curve of adipose tissue to bright light exposure. **A** Detrended bioluminescence trace of adipose explant from PER2::LUC mice following either 4-h incubation on a white light box (LIGHT) at 25 h, 31 h, 37 h or 43 h after the last 5% serum-media change or 4 h incubation in the dark (DARK), *n* ≤ 4 biological replicates (dishes of tissue from 8 mice). The data shown were used in the change of phase quantification. Adipose explants were cultured in 1 µM all-trans-retinal. **B** Quantification of treatment-induced change (Δ) in phase (h) from (**A**), *n* = 4, mean ± SEM, one-way ANOVA, ns = 0.933. blue = DARK, dark purple = T25 LIGHT, purple = T31 LIGHT, magenta = T37 LIGHT, pink = T43 LIGHT. **C** Phase-response curve derived from light conditions in (**B**), mean ± SEM.

In the present study, we found no effect of light exposure on the circadian clock in murine scWAT. This lack of response may not be surprising given that nocturnal mice experience limited evolutionary pressure for scWAT clock entrainment due to their nighttime activity, light-impermeable fur, and the protected localization of their scWAT depots. Despite this, opsins in murine scWAT remain light-sensitive and can influence adipocyte metabolism^17–19^. Importantly, similar independence from the circadian clock has been observed in other tissues where direct activation of opsins influences physiological processes, such as in the hypothalamus, blood vessels, and skin^24^. For example, blue light-mediated photorelaxation of smooth muscle in various organs leads to vasodilation and thereby reduced blood pressure^16,25^. It is possible that these light-responsive signaling pathways still function in synergy with circadian mechanisms, where the effects of opsin activation might vary with the time of day, thus supporting metabolic homeostasis. In this context, we recognize that the impact of feeding and endocrine signaling is very likely to play a greater role in scWAT metabolism than light. Yet, the potential interplay in vivo between light exposure, feeding, and hormonal regulation warrants further investigation. Future studies should explore the differential metabolic impact of diurnal versus nocturnal opsin activation in rodent and human scWAT in conjunction with time-of-day-associated changes in feeding and endocrine signaling. Disruptions to these pathways–such as those from artificial light exposure at night or shift work–could contribute to the development of metabolic disorders.

While our results indicate that the adipose tissue clock does not entrain to light stimulation in vitro, other tissues have been reported to exhibit local circadian photoentrainment. For example, in *Opn4*^−/−^ mice, which are unable to behaviorally entrain to light–dark cycles, the skin-clock gene expression phase remains synchronized to the light–dark cycle, whereas other peripheral clocks remain free-running^26^. Neuropsin (OPN5) has been shown to mediate local, light-dependent effects on circadian clock genes, supporting photoentrainment in murine outer ear skin exposed to light^26^. Additionally, OPN5 is required for light-driven entrainment of circadian rhythms in the retina and cornea ex vivo, independently of the SCN^27^.

In summary, the absence of a phase shift in response to bright white light exposure in scWAT in the present study suggests that light modulates adipocyte metabolism independently of any action on its circadian phase. However, it remains unknown to what extent human scWAT might be stimulated through the skin in vivo by naturally occurring light intensities and how this could influence scWAT metabolism. Such clinical studies are particularly relevant given the current “indoor lifestyle” in Western societies, in which people spend 80–90% of their time indoors^28–30^. Indoor environments generally provide constant artificial lighting, which results in inadequate light exposure during the day and excessive exposure in the evening compared to natural outdoor conditions^31–33^. Furthermore, the widespread use of clothing limits light exposure to most skin areas, thereby restricting daylight’s ability to reach scWAT. Due to the lack of daylight through the skin, daily metabolic regulation of scWAT could be impaired.

## Methods

### Animals

PER2::LUC mice (originally supplied by Joe Takahashi, University of Texas Southwestern) were housed in a specified pathogen-free barrier facility. For husbandry and non-experimental housing, mice were group-housed with environmental enrichment under 12 h:12 h light:dark cycles. All animal experiments were licensed under the 1986 Home Office Animal Procedures Act (UK) and carried out in accordance with local animal welfare committee guidelines.

### Adipose explant bioluminescence assay

Five male (for the initial experiments) and eight male or female (for the PRC experiments) PER2::LUC mice were euthanised by cervical dislocation and confirmed by exsanguination (conducted consistently at 09:00AM). Subcutaneous white adipose tissue was dissected from mice and kept on ice in 1× Hank’s Buffered Salt Solution (HBSS) (Gibco, 14025092) until plating at 10:00AM. Adipose tissue was cut into 1–3 mm pieces on ice in 1× HBSS and placed onto Millicell culture membranes (Millipore, #PICMORG50) within 35 mm dishes. 1.2 mL of LumiCycle recording media, DMEM (Gibco, 31053), 1× Glutamax (Gibco, 35050061), 20 mM MOPS (Sigma-Aldrich, M1442) 1 mM sodium pyruvate (Gibco, 11360070), 5% Hyclone III serum (SH30109.03), 100 units/mL and 100 µg/mL penicillin/streptomycin, 1 mM D-Luciferin potassium salt (Biosynth, #L-8220), 350 mOsm, pH 7.6, was added to dishes. Dishes were vacuum-sealed with silicone grease (CHT Silicones, SG-M494), 40 mm round coverslips (VWR, 631-0177), and transferred to a LumiCycle (ActiMetrics). Following a pre-recording period of at least 24-h, dishes were transported in a dark box to an incubator containing a white light box. Light-treated dishes were incubated on the white light box, illuminated with 6 Luxeon 3020 LED lights for 4-h. Light intensity (approximately 1800 photopic lux) was assessed using a digital lux meter (TENMA, TEN01070) held 0.25 cm above the white light box, at the approximate distance the cell cultures were placed from the light source. In this context, the maximal white light-induced inward current in cultured mouse adipocytes was previously shown to occur at ~450–480 nm blue light^17^. ‘Dark’, vehicle (DMSO), and corticosterone (CORT)-treated groups were incubated for 4-h in a dark box.

Following light or dark incubation, dishes were immediately transferred back into the LumiCycle, continuing with bioluminescence recording for a further 5 days. In the initial experiments, dishes were incubated with light approximately 2-h after the peak of PER2::LUC oscillation, and 31-h after the last media change. Prior to dark incubation, dishes were treated with 100 nM CORT or DMSO. In the PRC-yielding experiments, either 25, 31, 37, or 43-h after the last media change, dishes were incubated in light or dark for 4-h without any pharmacological or solvent intervention.

### Data acquisition and analysis

Bioluminescence intensity was recorded every 6 min with one of four photomultiplier tubes (PMTs) as counts per second (CPS), denoted relative luminescence units (RLU). Raw data from the LumiCycle bioluminescence recording was exported into Microsoft Excel from ActiMetrics software. The first 24-h after perturbation to cells was excluded from analysis to account for the transient effects of drug treatments on the clock gene-based bioluminescence reporter. To quantify circadian phase, data were detrended using a 24-h moving average in Excel and analyzed in GraphPad Prism v10.1. Detrended traces were fitted to a damped cosine wave using the following equation:

$$y=({mx}+c)+{{ae}}^{-{kx}}\cos \left(\frac{2{\pi }x-r}{p}\right)$$

Where *y* is the signal, *x* the corresponding time, a is amplitude i.e., the peak height of the waveform above the trend line, *k* the decay constant (1/*k* is the half-life), *r* the phase in radians and p the period. Period and phase are objectively quantified as the time taken for a cycle to occur and the shift relative to a cos wave, respectively. Area under the curve analysis was performed on the fitted data to generate X values for each peak. Phase-shift in hours, Δ phase (h) was calculated by subtracting the mean time of the first peak 24-h after the final perturbation for the untreated or dark (DARK) control condition from the first peak 24-h after the final perturbation for each technical replicate of the experimental conditions. For statistical analysis, a one-way ANOVA was conducted to evaluate the effects of various in vitro conditions on circadian phase. Following the ANOVA, Holm–Sidak’s post hoc multiple comparisons tests were performed to identify specific pairwise differences between the conditions.

## Data Availability

All data can be provided upon reasonable request to the corresponding author.
